# Density games^☆^

**DOI:** 10.1016/j.jtbi.2013.05.029

**Published:** 2013-10-07

**Authors:** Sebastian Novak, Krishnendu Chatterjee, Martin A. Nowak

**Affiliations:** a IST Austria, Am Campus 1, 3400 Klosterneuburg, Austria; b Program for Evolutionary Dynamics, Department of Mathematics, Harvard University, Cambridge, MA 02138, USA; c Program for Evolutionary Dynamics, Department of Organismic and Evolutionary Biology, Harvard University, Cambridge, MA 02138, USA

**Keywords:** Evolutionary game theory, Evolutionary dynamics, Replicator equation, Carrying capacity, Mathematical biology

## Abstract

The basic idea of evolutionary game theory is that payoff determines reproductive rate. Successful individuals have a higher payoff and produce more offspring. But in evolutionary and ecological situations there is not only reproductive rate but also carrying capacity. Individuals may differ in their exposure to density limiting effects. Here we explore an alternative approach to evolutionary game theory by assuming that the payoff from the game determines the carrying capacity of individual phenotypes. Successful strategies are less affected by density limitation (crowding) and reach higher equilibrium abundance. We demonstrate similarities and differences between our framework and the standard replicator equation. Our equation is defined on the positive orthant, instead of the simplex, but has the same equilibrium points as the replicator equation. Linear stability analysis produces the classical conditions for asymptotic stability of pure strategies, but the stability properties of internal equilibria can differ in the two frameworks. For example, in a two-strategy game with an internal equilibrium that is always stable under the replicator equation, the corresponding equilibrium can be unstable in the new framework resulting in a limit cycle.

## Author-Highlights

• We characterize a density-dependent game theoretical dynamics.
• Payoffs determine carrying capacities of phenotypes.
• We demonstrate similarities and differences between our framework and the classical approach.
• For certain parameter combinations, limit cycles can emerge.

## 1 Introduction

Evolutionary game theory studies frequency dependent selection. The fitness values of different phenotypes are not constant but depend on the frequencies of phenotypes in the population (Hofbauer and Sigmund, 1988, 1998, 2003; Maynard Smith, 1982; Nowak, 2006; Traulsen et al., 2007; Weibull, 1997). Reproductive success is often a linear function of the frequencies. The coefficients in this function are the entries of the payoff matrix. Evolutionary game theory was introduced as a framework for studying animal behavior (Maynard Smith, 1979; Maynard Smith and Price, 1973), but in the meanwhile has been extended to a wide array of applications ranging from viruses to humans (Archetti and Scheuring, 2011; Bshary et al., 2008; Damore and Gore, 2012; Doebeli and Knowlton, 1998; Dreber et al., 2008; Dugatkin and Reeve, 1998; Fowler and Christakis, 2010; Fu et al., 2008; Helbing, 2011; Milinski, 1987; Nowak et al., 2010; Ostrom, 1990; Pfeiffer et al., 2001; Rand et al., 2009; Rockenbach and Milinski, 2006; Turner and Chao, 1999; Wedekind and Milinski, 2000). There is much fruitful interaction between evolutionary and economic game theory (Alger and Weibull, 2010; Berger, 2011; Bergstrom et al., 1986; Binmore, 1994; Camerer, 2003; Cressman, 2003; Fudenberg and Tirole, 1991; Harsanyi and Selten, 1988; Nowak et al., 2004; Osborne and Rubinstein, 1994; Samuelson, 1997; Sigmund, 2010; Sigmund et al., 2010; Skyrms, 1996; Weibull, 1997). Constant selection, which is a special case, describes how populations adapt on constant fitness landscapes (see for example Eigen and Schuster, 1977). In evolutionary game theory the fitness landscape changes as the population moves over it (Nowak and Sigmund, 2004).

The standard approach for studying deterministic evolutionary game dynamics in infinitely large, well-mixed populations is the replicator equation (Hofbauer et al., 1979; Hofbauer and Sigmund, 1988; Taylor and Jonker, 1978; Zeeman, 1981). The basic assumption is that the payoff from the game determines the reproductive rate of individuals. The total population size is held constant by a variable death rate. The replicator equation for *n* strategies is given by

(1) $${\overset{˙}{y}}_{i}=y_{i}(f_{i}-\overset{¯}{f})\;i=1,\ldots ,n.$$

Here, *y*_*i*_ is the frequency of strategy *i* and ${\overset{˙}{y}}_{i}$ is the time derivative of this quantity. The payoff of strategy *i* is $f_{i}=\sum_{j=1}^{n}a_{\mathrm{ij}}y_{j}$. The coefficients $a_{\mathrm{ij}}\in R$ are the entries of the *n*×*n* payoff matrix with *a*_*ij*_ denoting the payoff for strategy *i* when interacting with strategy *j*. The average payoff of the population is $\overset{¯}{f}=\sum_{j=1}^{n}f_{j}y_{j}$. We have $\sum_{i=1}^{n}y_{i}=1$ at all times. Thus, the replicator equation is defined on the simplex *S*_*n*_. The interior of the simplex and all its faces are invariant under the replicator dynamics.

Hofbauer and Sigmund (1988) proved that a replicator equation for *n* strategies is equivalent to a Lotka–Volterra equation for n−1 species, thereby proving an interesting link between evolutionary game theory and a fundamental equation of theoretical ecology (May, 1973; Okubo and Levin, 2002).

An important consideration in ecological models is density limiting effects. Reproductive rates can depend on population size. A typical idea is that the reproductive rates decline as the population size increases. In the game-theoretical context, this concept was studied in, e.g., Cressman (1990a), Cressman (1990b), Cressman (1992), Cressman and Dash (1987), and Cressman and Garay (2003)). In this paper, we focus on a concrete case of density-dependent growth functions where the payoff from the game affects the susceptibility of individuals to density limiting effects. The association between payoff and density limitation seems entirely natural: successful individuals (with high payoff) may be stronger in fighting off competitors, may be more resistant to adverse effects of crowding, may thrive on lower energy supply, or may be more efficient in utilizing resources for reproduction.

We propose to study the evolutionary dynamics of the following equation:

(2a) $${\overset{˙}{x}}_{i}=r_{i}x_{i}(1-\frac{x_{T}}{K_{i}})\;i=1,\ldots ,n.$$

Here, *x*_*i*_ is the abundance of strategy *i* and ${\overset{˙}{x}}_{i}$ is its time derivative. The total population size, $x_{T}=\sum_{i=1}^{n}x_{i}$, is not constant, the parameters $r_{i}>0$ denote the net reproductive rates of phenotype *i* in the absence of density limitation, and *K*_*i*_ describes the carrying of capacity phenotype *i*. We assume that the *r*_*i*_ parameters are constant, while the *K*_*i*_ parameters depend on the frequencies and the payoffs as follows:

(2b) $$K_{i}=\overset{n}{\underset{j=1}{\sum }}a_{\mathrm{ij}}\frac{x_{j}}{x_{T}}.$$

Note that $y_{j}=x_{j}/x_{T}$ is the frequency of strategy *j* in the population. Similar to before, $a_{\mathrm{ij}}>0$ is the payoff for strategy *i* versus *j*, but we require these values to be positive such that the *K*_*i*_ can be interpreted as carrying capacities.

In our system, we want to separate growth rates (at low abundance) and carrying capacities (equilibrium abundances); the payoff from the game only affects the latter, but not the former. In addition, the *r*_*i*_ values do not affect equilibrium abundances. Furthermore, the dynamics given by Eqs. (2) has the property that, in isolation, phenotype *i* has carrying capacity $a_{\mathrm{ii}}>0$, and — since $r_{i}>0$ — that all trajectories are driven away from zero, so the population does not go extinct. In the following, we will call the origin, *x*_*i*_=0 for all *i*, the *trivial* equilibrium of Eqs. (2). All other equilibria will be denoted $60#?tjl$62#?>as *non-trivial* equilibria. *Monomorphic* equilibria are equilibria with exactly one strategy present, i.e., exactly one *x*_*i*_ is positive, and *internal* equilibria have all strategies present, i.e., $x_{i}>0$ for all *i*.

Our main results are as follows:

1. We show that the equilibria and the stability conditions of the monomorphic equilibria in the new model, Eqs. (2), coincide to those of the well-known replicator equation dynamics, Eq. (1), apart from the additional, trivial equilibrium *x*_*i*_=0 for all *i*, which is unstable.
2. For two strategies (*n*=2) with equal growth rates ($r_{1}=r_{2}$) we show that, along with the equilibria and the stability conditions of the monomorphic equilibria, the stability analysis of the internal equilibrium also coincides with the replicator equation dynamics.
3. For two strategies with unequal growth rates ($r_{1}\neq r_{2}$), the analysis differs from the replicator equation dynamics. While the equilibria and the stability conditions of the monomorphic equilibria are the same, the stability analysis of the internal equilibria is different. For example, in a two-strategy game with an internal equilibrium that is always stable in the replicator equation, the corresponding equilibrium can be unstable in our new framework resulting in a limit cycle. Furthermore, we present a complete characterization of the stability analysis for the two-strategy case based on the trace and the determinant of the Jacobian at the internal equilibrium.

## 2 Equilibria and stability conditions of monomorphic equilibria

In this section, we consider the model described by Eqs. (2) for *n* strategies and show that the equilibrium densities do not depend on the growth rates *r*_*i*_. Furthermore, strategy frequencies at non-trivial equilibria and stability conditions of the monomorphic equilibria are identical to those known from the replicator equation. In particular, the stability conditions of the monomorphic equilibria are independent of the growth rates *r*_*i*_.

*Equilibria characterization*. For the equilibria characterization, we reformulate Eqs. (2) as

(3) $${\overset{˙}{x}}_{i}=\frac{r_{i}}{K_{i}}x_{i}(K_{i}-x_{T})\;i=1,\ldots ,n.$$

We see from Eq. (3) that all strategies present at equilibrium have the same payoff, i.e., $K_{i}=x_{T}$ for every strategy *i* with non-zero abundance. Thus, at equilibrium all payoffs are equal to the average payoff, which is the exact same condition as for the replicator equation. Therefore, the number of non-trivial equilibria and the relative strategy frequencies at equilibrium are identical for both dynamics, Eqs. (1) and (2). In particular, equilibrium values are independent of *r*_*i*_.

*Stability of monomorphic equilibria*. We now show that the linear stability conditions for monomorphic equilibria are identical to those known from the replicator equation (and hence, independent of *r*_*i*_). A monomorphic equilibrium is given by $E_{i}=a_{\mathrm{ii}}e_{i}$, where $e_{i}$ is the *i*-th unit vector. With only one strategy present (i.e., *n*=1), *E*_1_ is a stable population size for the one-dimensional dynamics Eqs. (2). The Jacobian matrix at *E*_*i*_ is a triangular matrix, hence its eigenvalues can be read from its diagonal. The *j*-th diagonal entry, and thus the *j*-th eigenvalue, is given by

(4) $$\{\begin{array}{cc} -r_{i} & \mathrm{if}\;j=i,\\ -r_{j}(\frac{a_{\mathrm{ii}}}{a_{\mathrm{ji}}}-1) & \mathrm{if}\;j\neq i. \end{array}$$

The *i*-th eigenvalue reflects the fact that *E*_*i*_ is a stable population size if only strategy *i* is present. The remaining eigenvalues assert that strategy *j* cannot invade at *E*_*i*_ if $a_{\mathrm{ii}}>a_{\mathrm{ji}}$. Therefore, *E*_*i*_ is asymptotically stable if $a_{\mathrm{ii}}>a_{\mathrm{ji}}$ for all j≠i.

We summarize our results in the following theorem.

Theorem 1 **Equilibria and stability of monomorphic equilibria**

*Consider the evolutionary dynamics for* n≥2 *strategies given by Eqs.* (2):

(i) *The number of non-trivial equilibria and the relative strategy frequencies at any non-trivial equilibrium are identical to those known from the replicator equation*.
(ii) *The monomorphic equilibrium* $x_{i}>0$, *x*_*j*_=0 *for* j≠i, *is asymptotically stable if* $a_{\mathrm{ii}}>a_{\mathrm{ji}}$ *for every strategy* j≠i.

Note that Maynard Smith defined an evolutionarily stable strategy (ESS) of a game as “a strategy such that, if all the members of a population adopt it, no mutant strategy can invade” (Maynard Smith, 1982). His definition is stationary in the sense that it is based on a payoff matrix and hence is independent of any dynamics. If $P(S_{1},S_{2})$ denotes the payoff of strategy *S*_1_ against strategy *S*_2_, then *S*^⁎^ is evolutionarily stable if

$$P(S^{⁎},S^{⁎})>P(S,S^{⁎})\;\mathrm{or}\;P(S^{⁎},S^{⁎})=P(S,S^{⁎})\;\mathrm{and}\;P(S^{⁎},S)>P(S,S)$$

for all strategies *S* different from *S*^⁎^. In particular, this definition contains the case where the linearization around equilibria has vanishing eigenvalues and includes invasion by mixed strategies. Therefore, we only deal with asymptotic stability of equilibria of our dynamics, Eqs. (2).

It can be shown that every ESS is an asymptotically stable equilibrium of the replicator equation, Eq. (1). Conversely, not every asymptotically stable equilibrium is an ESS. For games with density dependent payoffs, $a_{\mathrm{ij}}=a_{\mathrm{ij}}(x_{T})$, the notion of a density dependent evolutionarily stable strategy (DDESS) exists (Cressman, 1990a,b) and has been extended to nonlinear payoff functions (Cressman, 1988). Similarly to the density-independent case, there is a strong relationship between a DDESS and an asymptotically stable equilibrium of the dynamics ${\overset{˙}{x}}_{i}=x_{i}f_{i}(x_{T})$, where $f_{i}(x_{T})=\sum_{j=1}^{n}a_{\mathrm{ij}}(x_{T})x_{i}/x_{T}$. However, our model is structurally different since payoffs determine carrying capacity instead of reproductive rate. A characterization of evolutionarily stable strategies for our dynamics, Eqs. (2), will be considered in future work.

## 3 Two strategies with equal growth rates

In this section, we consider the case of two strategies with equal growth rates. We first illustrate the result of Theorem 1 in this special case below. Set *n*=2, $r_{1}=r_{2}=r$, and write the payoff matrix as A=(acbd). Then, solving for internal equilibria produces

x^1=(d−b)(ad−bc)(a−c+d−b)2,x^2=(a−c)(ad−bc)(a−c+d−b)2,

x^T=K1=K2=ad−bca−c+d−b.

Thus, y^1=x^1/x^T=(d−b)/(a−c+d−b). From Theorem 1, we conclude that the monomorphic equilibrium with only strategy 1 present is asymptotically stable if a>c, or *a*=*c* and b>d, and similarly for strategy 2. It is easy to see that the internal equilibrium exists if a≠c, d≠b, and sgn(a−c)=sgn(d−b). Straightforward linear stability analysis shows that it is an attractor if this sign is negative. Because there is an invariant line connecting the trivial equilibrium (0,0) with the internal equilibrium, we can exclude the existence of limit cycles and hence the attractor is global. The system is bistable for a−c>0 and d−b>0. Therefore, equilibrium frequencies and stability conditions in that case match those from the replicator equation (Hofbauer and Sigmund, 1998).

Another simple calculation shows that for any given Δ∈(0,∞), the per-capita growth rate ${\overset{˙}{y}}_{i}$ does not change its sign along the line $x_{2}=\Delta x_{1}$ and equals zero along $x_{2}/x_{1}=(a-c)/(d-b)$; thus, the line connecting the origin (0,0) with the internal fixed point (given its existence) is invariant. This indicates that the two-dimensional dynamics can be projected on one dimension without losing the essential information—indeed, the dynamics for the frequencies reduces to the one-dimensional problem

(5) $${\overset{˙}{y}}_{1}=y_{1}y_{2}(K_{1}-K_{2})C_{1}.$$

**Fig. 1:**
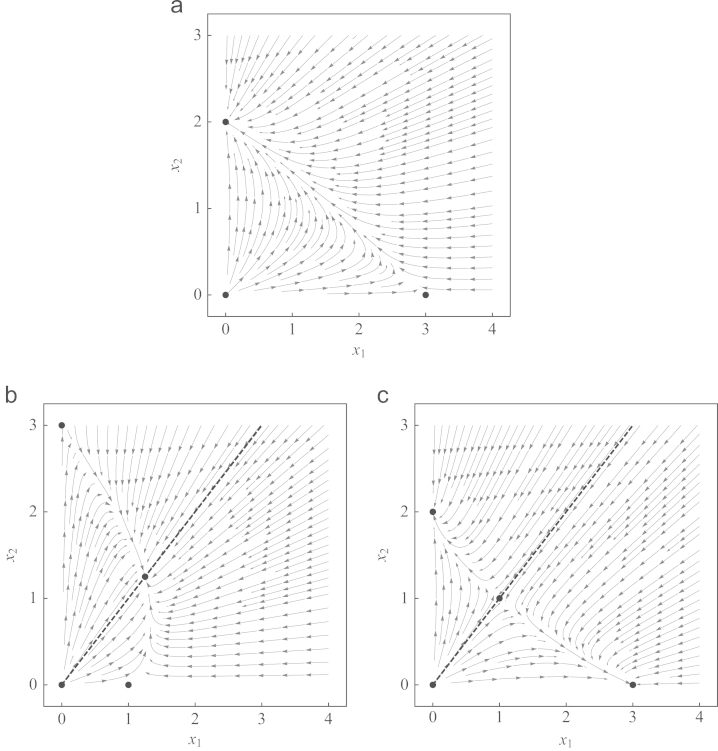
Phase portraits of classical games with $r_{1}=r_{2}=1$. In the Prisoners' dilemma, (a), strategy 2 (defection) dominates strategy 1 (cooperation). In the Hawk–Dove game, (b), the two strategies coexist. The Stag hunt game, (c), is bistable. The dashed lines in (b) and (c), given by $x_{2}/x_{1}=(a-c)/(d-b)$, are invariant under the dynamics.

The expression $C_{1}={\mathrm{rx}}_{T}/(K_{1}K_{2})$ is always positive; the standard replicator equation corresponds to the case $C_{1}\equiv 1$. Therefore, Eq. (5) behaves exactly like a replicator equation apart from the fact that the speed of the trajectories is modified by the influence of population density *x*_*T*_. Consequently, phase portraits behave as expected, see Fig. 1. In the Prisoners' dilemma, defection wins over cooperation (Fig. 1a), in the Hawk–Dove game, the two strategies coexist (Fig. 1b), and in the Stag hunt game, the system is bistable (Fig. 1c).

Theorem 2 **Two strategies with equal growth rates**

*Consider the evolutionary dynamics given by Eqs.* (2) *for n*=2 *strategies with* $r_{1}=r_{2}$. *In addition to the statements in Theorem* 1, *the stability conditions for the internal equilibrium are identical to those from the replicator equation. The projection of the dynamics on strategy frequencies*, *Eq*. (5), *exhibits the same equilibria and stability conditions as the replicator equation dynamics*, *Eq*. (1).

Note that it is standard to rewrite any system of the form ${\overset{˙}{x}}_{i}=x_{i}F_{i}(x_{1},\ldots ,x_{n})$ in terms of frequencies and total population size (Hofbauer and Sigmund, 1998), i.e., to split up the dynamics into *evolutionary* and *ecological* dynamics (Cressman and Garay, 2003). The expression Eq. (5) is the evolutionary component of our dynamics, Eqs. (2). The ecological component reads

$${\overset{˙}{x}}_{T}=x_{T}\overset{2}{\underset{i=1}{\sum }}{\mathrm{ry}}_{i}(1-\frac{x_{T}}{K_{i}}),$$

but can be omitted since it does not critically influence the frequency dynamics. This is not so straightforward in the case of more strategies or unequal growth rates $r_{i}\neq r_{j}$, as we will see in the following section.

## 4 Two strategies with unequal growth rates

In this section we consider the general case of two strategies, *n*=2, but the growth rates *r*_1_ and *r*_2_ are not equal. By the results of Section 2, we know that the equilibria and the stability conditions of monomorphic equilibria coincide with the well-known replicator equation. We will focus on the stability of internal equilibria and present a complete characterization of the stability analysis which shows a contrast as compared to the replicator equation.

### 4.1 The effect of different growth rates

**Fig. 2:**
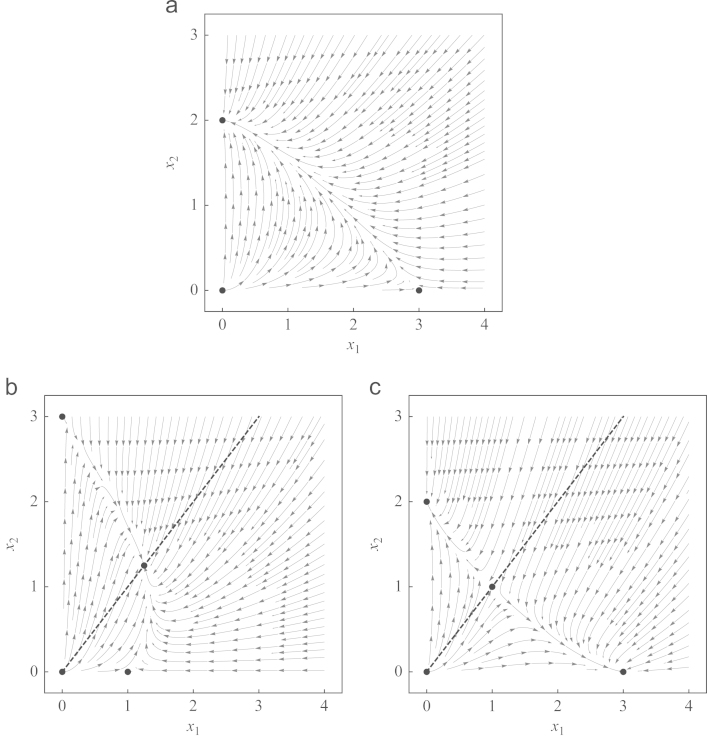
Phase portraits of classical games with $r_{1}=1$ and $r_{2}=2$. The payoff values and the qualitative behavior in (a) Prisoners' dilemma, (b) Hawk–Dove game, and (c) Stag hunt game are the same as in Fig. 1. However, the trajectories are different and the dashed lines in (b) and (c), given by $x_{2}/x_{1}=(a-c)/(d-b)$, are not invariant as in Fig. 1.

In the general case, when the growth rates are different ($r_{1}\neq r_{2}$) the picture is different from the special case of equal growth rates considered in Section 3. As shown in Section 2, equilibria and stability conditions of monomorphic equilibria remain unchanged, independent of *r*_1_ and *r*_2_. Nevertheless, we cannot reduce our model to a single equation as we did in Section 3 (to Eq. (5)), since the sign of the change in strategy frequencies depends on the absolute population size. In other words, the relative per-capita growth rate, ${\overset{˙}{y}}_{i}$, can change its sign along straight lines, $x_{2}=\kappa x_{1}$ (κ>0), as can be seen in Fig. 2. Accordingly, the projection on relative frequencies to obtain the *evolutionary dynamics* (Cressman and Garay, 2003, see above) reveals an analogue of the replicator equation with nonlinear payoffs that depend on the population size *x*_*T*_

$${\overset{˙}{y}}_{i}=y_{1}y_{2}(r_{1}(1-\frac{x_{T}}{K_{1}})-r_{2}(1-\frac{x_{T}}{K_{2}})).$$

**Fig. 3:**
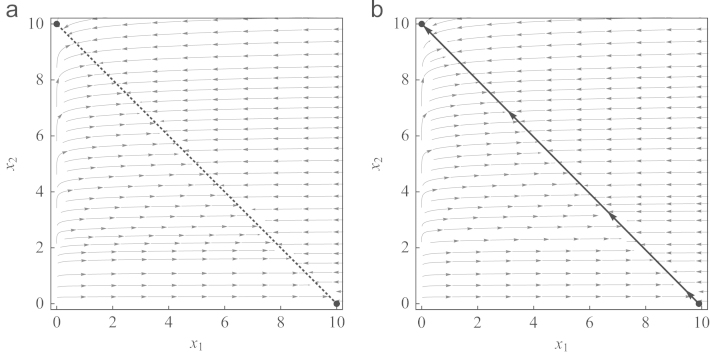
With very disparate growth rates, essentially only the fast-growing strategy changes its abundance until carrying capacity is reached. At carrying capacity, the difference in growth rates becomes ineffective, such that the structure of the payoff matrix, *A*, determines the dynamics. (a) If all payoffs are the same, $a_{11}=a_{12}=a_{21}=a_{22}=10$, then the dotted line $x_{1}+x_{2}=10$ is a continuum of equilibria. Thus, starting with an initial population composition, *x*_1_ remains more or less constant and *x*_2_ adjusts such that carrying capacity is reached—given that *x*_1_ is not too low and *x*_2_ not too high, initially. (b) If the complete symmetry in the payoffs is broken, $a_{11}=9.9$ and $a_{12}=a_{21}=a_{22}=10$, all trajectories move to a slow manifold (bold line) close to $x_{1}+x_{2}=10$ relatively quickly. Trajectories are nearly horizontal since *x*_1_ grows much faster than *x*_2_. At this manifold, they slowly converge to the global attractor (0,10), because the payoff configuration favors strategy 2.

As an example, consider a game with uniform payoffs, $a_{11}=a_{12}=a_{21}=a_{22}=a$. Then, every point on $x_{1}+x_{2}=a$ is an equilibrium, no matter how growth rates are chosen. For *a*=10, $r_{1}=50$, and $r_{2}=1$, the corresponding phase portrait is depicted in Fig. 3a. It shows that even with very disparate growth rates the stability properties of the pure equilibria cannot be changed in the degenerate case $a_{\mathrm{ij}}\equiv \mathrm{const}$. Thus, the effect of a large discrepancy in growth rates is neutral with respect to equilibria, but leads to nearly horizontal trajectories in strategy density space, such that effectively only the fast-growing strategy changes its abundance when the dynamics converges to a continuum of equilibria. However, the slightest change in payoffs breaks the symmetry, such that the curve of equilibria collapses and the equilibrium with the higher payoff is approached, see Fig. 3b. Trajectories move towards a slow manifold in a short initial phase, during which strategy 2 hardly changes in abundance. When population size is saturated, the difference in growth rates becomes effective, such that strategy 1 is able to out-compete strategy 2 (compare the concepts of *r*- and *K-selection*, MacArthur and Wilson, 1967). Thus, even a highly increased growth rate cannot make up for a slightly worse payoff in the long run.

### 4.2 The internal equilibrium

In this section, we consider the case that a unique internal fixed point exists, i.e., the expressions a−c and d−b have the same sign (it follows that the sign of ad−bc is the same as that of a−c and d−b). Note that under the replicator equation, the internal fixed point is the global attractor if a−c<0 and d−b<0, and the system is bistable if a−c>0 and d−b>0 (Hofbauer and Sigmund, 1998).

*Notations: Characteristic polynomial of the Jacobian*. For the internal equilibrium, we calculate the characteristic polynomial, *g*, of the Jacobian matrix at the internal fixed point, *J*

$$g(\lambda )=\lambda^{2}-\mathrm{tr}(J)\cdot \lambda +\det(J),$$

where the *trace* tr(*J*) and the *determinant* det(*J*) are as follows:

$$\mathrm{tr}(J)=-\frac{(d-b)\alpha_{1}r_{1}+(a-c)\alpha_{2}r_{2}}{(a-c+d-b)(\mathrm{ad}-\mathrm{bc})},\det(J)=-\frac{(a-c)(d-b)r_{1}r_{2}}{\mathrm{ad}-\mathrm{bc}},$$

where

$$\alpha_{1}=\mathrm{ad}-\mathrm{bc}+(a-c)(b-a),\alpha_{2}=\mathrm{ad}-\mathrm{bc}+(d-b)(c-d).$$

We omit the expression of the matrix *J* since it is not needed here and its derivation is straightforward. According to the Routh–Hurwitz criterion (Hurwitz, 1895; Routh, 1877), an internal equilibrium x^ is stable if tr(J)<0 and det(J)>0. Obviously, det(J)>0 if a−c<0 and d−b<0 (hence also ad−bc<0). Therefore, the critical quantity is tr(*J*).

*Analysis of tr*(*J*). For a given payoff matrix, we interpret $\mathrm{tr}(J)=\mathrm{tr}(J)(r_{1},r_{2})$ as a function of the growth rates $r_{1}>0$ and $r_{2}>0$. Straightforward calculations show that tr(J)(0,0)=0 and the derivatives are

(6a) $$\frac{\partial \;\mathrm{tr}(J)(r_{1},r_{2})}{\partial r_{1}}=-\frac{(d-b)\alpha_{1}}{(a-c+d-b)(\mathrm{ad}-\mathrm{bc})},$$

(6b) $$\frac{\partial \;\mathrm{tr}(J)(r_{1},r_{2})}{\partial r_{2}}=-\frac{(a-c)\alpha_{2}}{(a-c+d-b)(\mathrm{ad}-\mathrm{bc})}.$$

For fixed payoff values, these derivatives do not change their signs. Furthermore, we calculate

(7) $$\frac{\partial \;\mathrm{tr}(J)(r_{1},r_{2})}{\partial r_{1}}+\frac{\partial \;\mathrm{tr}(J)(r_{1},r_{2})}{\partial r_{2}}=-\frac{b(a-c)+c(d-b)}{(\mathrm{ad}-\mathrm{bc})}<0.$$

Thus, along the diagonal $r_{1}=r_{2}$, the function tr(*J*) is strictly decreasing and therefore negative for $r_{1}=r_{2}>0$.

For the analysis of tr(*J*), we have the following cases:

• *Case*1: $\alpha_{1}>0$ and $\alpha_{2}>0$.
If sgn(a−c)=sgn(d−b)=1, then tr(*J*) is negative for every choice of growth rates $r_{1},r_{2}>0$. The case that sgn(a−c)=sgn(d−b)=−1 cannot occur, since then both entries of $60#?tjl$62#?>the gradient of tr(*J*), Eqs. (6), are positive, which contradicts Eq. (7).
• *Case*2: $\alpha_{1}\alpha_{2}<0$.
If $\alpha_{1}$ and $\alpha_{2}$ have different signs, then the sign of tr(*J*) depends on the choice of *r*_1_ and *r*_2_, i.e., there are pairs of growth rates for which tr(*J*) has different signs. More precisely the sign of
  $$(d-b)\alpha_{1}r_{1}+(a-c)\alpha_{2}r_{2},$$
determines the sign of tr(*J*).
• *Case*3: $\alpha_{1}<0$ and $\alpha_{2}<0$.
The case that sgn(a−c)=sgn(d−b)=1 is not possible due to an argument analogous to the one in Case 1. If sgn(a−c)=sgn(d−b)=−1, then tr(*J*) is negative for every choice of growth rates $r_{1},r_{2}>0$.

Overall, we have shown:

Proposition 1 *Consider the evolutionary dynamics for n*=2 *strategies given by Eqs.* (2) *with the payoff matrix given by* A=(acbd), *such that an internal fixed point* x^ *exists*, *i.e.*, sgn(a−c)=sgn(d−b). *Then the following assertions hold*:

1. (*Determinant*). *The sign of the determinant*, *det*(*J*), *of the characteristic polynomial of the Jacobian at* x^ *is independent of the growth rates r*_1_ *and r*_2_. *If* a−c<0 *and* d−b<0, *then det*(*J*) *is positive*, *if* a−c>0 *and* d−b>0, *then det*(*J*) *is negative*.
2. (*Trace*). *Let* $\alpha_{1}=\mathrm{ad}-\mathrm{bc}+(a-c)(b-a)$ *and* $\alpha_{2}=\mathrm{ad}-\mathrm{bc}+(d-b)(c-d)$. *Then*, *we have the following characterization*:
  (i) *If* $\alpha_{1}\alpha_{2}>0$, *then the sign of* tr(*J*), *the trace of the characteristic polynomial of the Jacobian at* x^, *is negative*, *independent of the growth rates r*_1_ *and r*_2_ *and*
  (ii) *if* $\alpha_{1}\alpha_{2}<0$, *then*
    (a) tr(J)<0 *if* $(d-b)\alpha_{1}r_{1}+(a-c)\alpha_{2}r_{2}>0$ *and*
    (b) tr(J)>0 *if* $(d-b)\alpha_{1}r_{1}+(a-c)\alpha_{2}r_{2}<0$.

*Hence*, *the sign of* tr(*J*) *depends on the choice of r*_1_ *and r*_2_.

### 4.3 Interpretation of the results

In this section we analyze the case of two strategies with unequal growth rates and compare them to the dynamics of the well-studied replicator equation, see Hofbauer and Sigmund (1998). We will show the following:

• *Case* (i): a>c and d<b (or vice versa).
There is no internal fixed point under the replicator equation, strategy 1 (or strategy 2, in case of reversed inequalities) dominates over the other strategy. The same holds true for our model. All trajectories converge to the respective boundary equilibrium.
• *Case* (ii): a>c and d>b.
Under the replicator equation, the system is bistable. There is an unstable, internal fixed point and, depending on the initial condition, one strategy dominates the other. In our model, the same behavior can be observed, with the internal equilibrium being a saddle point. Apart from those starting on a separatrix connecting the origin with the internal fixed point (which is a straight line for equal growth rates $r_{1}=r_{2}$, see Section 3), all trajectories converge to one of the boundary equilibria. This is independent of the signs of $\alpha_{1}$ and $\alpha_{2}$, as argued below.
• *Case* (iii): a<c and d<b.
Under the replicator equation, the internal fixed point is asymptotically stable. In our model, the coexistence of the two strategies is guaranteed, but the situation is more complicated. The internal fixed point can lose stability and stable limit cycles can emerge (see below for the detailed analysis).

*Detailed analysis*. The fact that the trace of the Jacobian at the internal fixed point is the sum of its eigenvalues

$$\mathrm{tr}(J)=\lambda_{1}+\lambda_{2},$$

and that its determinant is the product of its eigenvalues

$$\det(J)=\lambda_{1}\lambda_{2},$$

allows for a more detailed analysis.

*Analysis of Case* (ii). In Case (ii), the determinant of the Jacobian at the internal equilibrium, det(*J*), is negative by Proposition 1. Hence, the eigenvalues of the Jacobian must have different signs and, in particular, they must be real (otherwise, they would be complex conjugates that have a positive product). Therefore, the internal equilibrium is a saddle point; it is not necessary to consider the trace tr(*J*) in this case.

*Analysis of Case* (iii) Assume that a<c and d<b, such that the determinant of the Jacobian at the internal fixed point, det(*J*), is positive. Therefore, the real parts of the eigenvalues of *J* have the same sign.

(a) If $\alpha_{1}\alpha_{2}>0$, then tr(J)<0 by Proposition 1 and hence both eigenvalues of *J* have negative real parts. Hence, the internal equilibrium is asymptotically stable.
(b) Now assume that $\alpha_{1}$ and $\alpha_{2}$ have different signs, $\alpha_{1}\alpha_{2}<0$.
  D1: If $(d-b)\alpha_{1}r_{1}+(a-c)\alpha_{2}r_{2}>0$, it is easy to see from the expression of tr(*J*) that tr(J)<0. Since the trace of the Jacobian is the sum of its eigenvalues, both eigenvalues have negative real parts. Therefore, the internal equilibrium x^ is asymptotically stable.
  D2: If $(d-b)\alpha_{1}r_{1}+(a-c)\alpha_{2}r_{2}<0$, then tr(J)>0. Hence, both eigenvalues of *J* have positive real parts and the internal equilibrium x^ is repelling.

When traversing from domain D1 into domain D2, both eigenvalues simultaneously cross the imaginary axis and neither vanishes, because det(*J*) is nonzero. Hence, a supercritical Hopf bifurcation occurs (Kuznetsov, 2004), which leads to an attracting limit cycle.

**Fig. 4:**
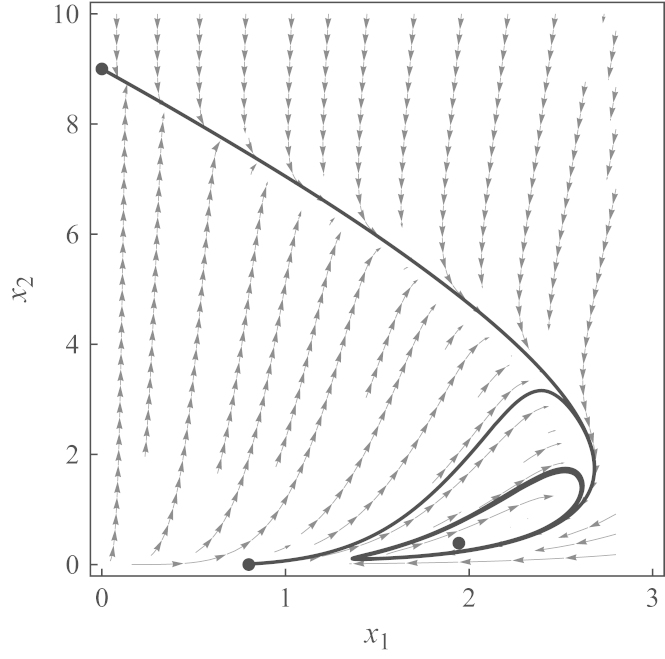
Phase portrait of Eqs. (2) for *a*=0.8, *b*=10, *c*=1, *d*=9, $r_{1}=1$, and $r_{2}=5$. All trajectories converge to an attracting limit cycle. Two exemplary trajectories (bold curves), starting near the monomorphic equilibria (0.8,0) and (0,9), were simulated. They approach a stable limit cycle around the internal equilibrium.

**Fig. 5:**
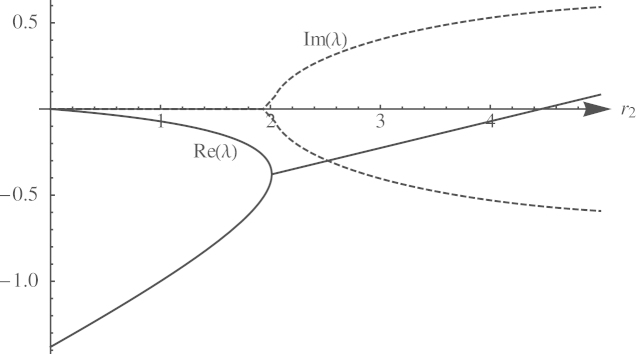
Eigenvalues of the internal equilibrium for *a*=0.8, *b*=10, *c*=1, *d*=9, $r_{1}=1$, and $r_{2}\in (1,5)$. The real parts of the eigenvalues are depicted by the solid curves, their imaginary parts by the dashed curves. Eigenvalues turn complex at $r_{2}\approx 2.01$, the Hopf bifurcation occurs at $r_{2}\approx 4.46$.

Example 1 An example of an attracting limit cycle is illustrated in Fig. 4. Fig. 5 shows the real parts (solid) and imaginary parts (dashed) of the eigenvalues along a path $\gamma (z)=({\overset{¯}{r}}_{1},z)$. First, they collide on the negative real axis and become complex, thereby transforming the internal equilibrium into an oscillatory attractor. Then, they cross the imaginary axis, turning the fixed point into a repellor and creating a limit cycle. This example also shows that indeed both scenarios, D1 and D2, are feasible: For instance, with *a*=0.8, *b*=10, *c*=1, *d*=9, $r_{1}=1$ and $r_{2}=2$, the internal equilibrium x^≈(1.94,0.39) is asymptotically stable, whereas with $r_{2}=5$, it is repelling (see Fig. 4).

In summary, we characterized the system of Eqs. (2) for two strategies:

Theorem 3 **Characterization for *n*=2**

*Consider the evolutionary dynamics for n*=2 *strategies given by Eqs.* (2), *let the payoff matrix be* A=(acbd) *and define*

$$\alpha_{1}=\mathrm{ad}-\mathrm{bc}+(a-c)(b-a),\alpha_{2}=\mathrm{ad}-\mathrm{bc}+(d-b)(c-d).$$

*Then, the dynamics can be characterized as follows*:

(i) *If either* a<c *and* d>b, *or* a>c *and* d<b, *then there is no internal fixed point; one strategy dominates the other.*
(ii) *If* a>c *and* d>b, *then the internal fixed point is a saddle point; the system is bistable.*
(iii) *If* a<c *and* d<b, *then the system is permanent, i.e., no strategy becomes extinct. There are two possibilities:*
  (a) $\alpha_{1}\alpha_{2}>0$: *The internal equilibrium is asymptotically stable for every choice of growth rates* $r_{1}>0$ *and* $r_{2}>0$.
  (b) $\alpha_{1}\alpha_{2}<0$:
    D1: *If* $(d-b)\alpha_{1}r_{1}+(a-c)\alpha_{2}r_{2}>0$, *the internal equilibrium is asymptotically stable.*
    D2: *If* $(d-b)\alpha_{1}r_{1}+(a-c)\alpha_{2}r_{2}<0$, *the internal equilibrium is a repellor and there is a stable limit cycle.*
    *Both cases, D1 and D2, can occur, as demonstrated in* Example1.

## 5 An alternative model

Our results derived in this paper, Proposition 1 and Theorems 1–3, are not unique to the proposed model, Eqs. (2). Consider the system

(8a) $${\overset{˙}{x}}_{i}=x_{i}(\frac{\beta_{i}}{1+\eta_{i}x_{T}}-1)\;i=1,\ldots ,n.$$

The parameters $\beta_{i}>1$ denote the birth rates of phenotype *i* in the absence of density limitation, death rates have been normalized to 1 for all strategies, and $\eta_{i}$ describes the effect of density limitation on phenotype *i*. We assume that the $\beta_{i}$ parameters are constant, while the $\eta_{i}$ parameters depend on frequencies and payoffs as follows:

(8b) $$\eta_{i}=\frac{\beta_{i}-1}{K_{i}},$$

where, as before, $K_{i}=\sum_{j=1}^{n}a_{\mathrm{ij}}x_{j}/x_{T}$. Note that Eqs. (8) is analogous to Eqs. (2) for a specific choice of density and payoff dependent growth rates.

For this model, the precise same statements from Theorems $60#?tjl$62#?>1–3 and Proposition 1 can be derived, and the phase portraits are very similar (results not shown). It is surprising that the conditions on the payoff values are identical for both models. In particular, the reappearance of the expressions $\alpha_{1}$ and $\alpha_{2}$, and the exact same conditions on their signs are worth noting. There is, however, a difference in the growth rate pairs that lead to limit cycles. For $60#?tjl$62#?>Eqs. (8) with a<c, d<b, and $\alpha_{1}\alpha_{2}<0$, the separatrix in $\beta_{1}-\beta_{2}-\mathrm{space}$, dividing configurations which exhibit limit cycles from those that do not, is given by a nonlinear equation (compare Case 2 in Section 4.2). Appendix A presents a more detailed analysis of this alternative model.

Overall, it is interesting to see that our results are not specific to a single model, and that games affecting carrying capacity can lead to unexpected behavior, namely the destabilization of internal equilibria.

## 6 Conclusion

The dominant assumption of evolutionary game theory of the last 40 years was that payoff affects reproductive rate: successful individuals are faster at producing offspring. But this is not the only possibility. In ecological and evolutionary processes there are other aspects of competition; an important one is density limitation.

In this paper we have studied a simple model, where the payoff from the game affects the exposure to density limiting effects. Successful individuals are less susceptible to density limitation. They thrive at larger population size, may be better at fighting off competitors, may resist the adverse affects of crowding, and may be able to grow more efficiently on lower food and energy supply. This extension of evolutionary game theory seems entirely natural and should have consequences that will affect both stochastic and spatial games (Antal et al. (2009a,b), Hauert et al., 2008; Imhof $60#?tjl$62#?>and Nowak, 2010; Killingback and Doebeli, 1996; Nowak and May, 1992; Nowak et al., 2004; Ohtsuki et al., 2006; Perc, 2009; Santos et al., 2006; Szabó and Fath, 2007; Tarnita et al., 2009; Van Veelen et al., 2012). In particular, it can be seen as an implementation of carrying capacity into the replicator dynamics. The comparison of different implementations, including exogenously fixed carrying capacities, will be considered for future work.

Here we have explored a deterministic, non-spatial system. We have found interesting similarities with the traditional replicator equation, but also important differences. For each non-trivial equilibrium of our equation there exists a corresponding equilibrium for the replicator equation, where each strategy has the same frequency and the same payoff. The linear stability conditions of pure strategies are the same for the two frameworks, but the stability conditions of internal equilibria can vary. Using our equation for a game where two strategies coexist, the internal equilibrium can become unstable resulting in limit cycles if the two strategies differ in their intrinsic reproductive rates.

## Appendix A Technical supplement

The model Eqs. (8) can be reformulated as

$${\overset{˙}{x}}_{i}=x_{i}(\beta_{i}-1)\frac{K_{i}-x_{T}}{K_{i}+(\beta_{i}-1)x_{T}}.$$

From this, it is obvious that the equilibria are identical to those from Eqs. (2). The Jacobian matrix at $E_{i}=a_{\mathrm{ii}}e_{i}$ is triangular, its diagonal entries being

$$\{\begin{array}{cc} -\frac{b_{i}-1}{b_{i}} & \mathrm{if}\;j=i,\\ -\frac{a_{\mathrm{ji}}(b_{j}-1)}{a_{\mathrm{ji}}+a_{\mathrm{ii}}(b_{j}-1)}(\frac{a_{\mathrm{ii}}}{a_{\mathrm{ji}}}-1) & \mathrm{if}\;j\neq i, \end{array}$$

where the expressions $(b_{i}-1)/b_{i}$ and $a_{\mathrm{ji}}(b_{j}-1)/(a_{\mathrm{ji}}+a_{\mathrm{ii}}(b_{j}-1))$ are positive. Hence, comparison to Eq. (4) shows that the linear stability conditions of the monomorphic equilibria are identical for the two models, Eqs. (2) and (8).

For two strategies and equal birth rates, $\beta_{1}=\beta_{2}=\beta$, the projection on strategy frequencies is

$${\overset{˙}{y}}_{1}=y_{1}y_{2}(K_{1}-K_{2})C_{2}.$$

Since $C_{2}=(\beta (\beta -1)x_{T})/[(1+\eta_{1}x_{T})(1+\eta_{2}x_{T})K_{1}K_{2}]$ is positive, the exact same conclusions from Section 3 hold true. If the growth rates are different, the projection is

$${\overset{˙}{y}}_{1}=y_{1}y_{2}(\frac{\beta_{1}K_{1}}{K_{1}+(\beta_{1}-1)x_{T}}-\frac{\beta_{2}K_{2}}{K_{2}+(\beta_{2}-1)x_{T}}).$$

Again, this can be interpreted as an analogue of the replicator equations with nonlinear payoffs that depend on population size *x*_*T*_.

For the stability analysis of the internal equilibrium, we obtain for the characteristic polynomial of the Jacobian at the internal fixed point, $\overset{˜}{J}$

$$g(\lambda )=\lambda^{2}-\mathrm{tr}(\overset{˜}{J})\cdot \lambda +\det(\overset{˜}{J}),$$

where

$$\mathrm{tr}(\overset{˜}{J})=-\frac{(a-c+d-b)[b(a-c)+c(d-b)]\beta_{1}\beta_{2}-(a-c)\alpha_{2}\beta_{1}-(d-b)\alpha_{1}\beta_{2}}{(a-c+d-b)(\mathrm{ad}-\mathrm{bc})\beta_{1}\beta_{2}},\det(\overset{˜}{J})=-\frac{(a-c)(d-b)(\beta_{1}-1)(\beta_{2}-1)}{(\mathrm{ad}-\mathrm{bc})\beta_{1}\beta_{2}},$$

with $\alpha_{1}=\mathrm{ad}-\mathrm{bc}+(a-c)(b-a)$ and $\alpha_{2}=\mathrm{ad}-\mathrm{bc}+(d-b)(c-d)$. In $60#?tjl$62#?>analogy to Section 4.2, the critical quantity is $\mathrm{tr}(\overset{˜}{J})$, which we interpret as a function of $\beta_{1}$ and $\beta_{2}$. Obviously, $\mathrm{tr}(\overset{˜}{J})(1,1)=0$. The gradient of $\mathrm{tr}(\overset{˜}{J})$ is given by

$$\frac{\partial \;\mathrm{tr}(\overset{˜}{J})(\beta_{1},\beta_{2})}{\partial \beta_{1}}=-\frac{(d-b)\alpha_{1}}{(a-c+d-b)(\mathrm{ad}-\mathrm{bc})\beta_{1}^{2}},\frac{\partial \;\mathrm{tr}(\overset{˜}{J})(\beta_{1},\beta_{2})}{\partial \beta_{2}}=-\frac{(a-c)\alpha_{2}}{(a-c+d-b)(\mathrm{ad}-\mathrm{bc})\beta_{2}^{2}},$$

and along the diagonal, $\beta_{1}=\beta_{2}=\beta$, we have

$$\frac{\partial \;\mathrm{tr}(J)(\beta ,\beta )}{\partial \beta_{1}}+\frac{\partial \;\mathrm{tr}(J)(\beta ,\beta )}{\partial \beta_{2}}=-\frac{b(a-c)+c(d-b)}{(\mathrm{ad}-\mathrm{bc})\beta^{2}}<0.$$

Thus, we recover the exact same cases as in Section 4.2; hence, Proposition 1, and therefore also Theorem 3 hold for Eqs. (8) with modified domains D1 and D2.
